# Unravelling the Secrets of Mycobacterial Cidality through the Lens of Antisense

**DOI:** 10.1371/journal.pone.0154513

**Published:** 2016-05-04

**Authors:** Parvinder Kaur, Santanu Datta, Radha Krishan Shandil, Naveen Kumar, Nanduri Robert, Upneet K. Sokhi, Supreeth Guptha, Shridhar Narayanan, Anand Anbarasu, Sudha Ramaiah

**Affiliations:** 1 Research Area, Drug Discovery, AstraZeneca India Private Limited, Bangalore, India; 2 Drug Discovery, Bugworks, C-CAMP, Bangalore, India; 3 Arthritis and Tissue Degeneration Program, Hospital for Special Surgery, New York, New York, United States of America; 4 School of Biosciences and Technology, VIT University, Vellore, India; Infectious Disease Research Institute, UNITED STATES

## Abstract

One of the major impediments in anti-tubercular drug discovery is the lack of a robust grammar that governs the in-vitro to the in-vivo translation of efficacy. *Mycobacterium tuberculosis* (Mtb) is capable of growing both extracellular as well as intracellular; encountering various hostile conditions like acidic milieu, free radicals, starvation, oxygen deprivation, and immune effector mechanisms. Unique survival strategies of Mtb have prompted researchers to develop in-vitro equivalents to simulate in-vivo physiologies and exploited to find efficacious inhibitors against various phenotypes. Conventionally, the inhibitors are screened on Mtb under the conditions that are unrelated to the in-vivo disease environments. The present study was aimed to (1). Investigate cidality of Mtb targets using a non-chemical inhibitor antisense-RNA (AS-RNA) under in-vivo simulated in-vitro conditions.(2). Confirm the cidality of the targets under in-vivo in experimental tuberculosis. (3). Correlate in-vitro *vs*. in-vivo cidality data to identify the in-vitro condition that best predicts in-vivo cidality potential of the targets. Using cidality as a metric for efficacy, and AS-RNA as a target-specific inhibitor, we delineated the cidality potential of five target genes under six different physiological conditions (replicating, hypoxia, low pH, nutrient starvation, nitrogen depletion, and nitric oxide).In-vitro cidality confirmed in experimental tuberculosis in BALB/c mice using the AS-RNA allowed us to identify cidal targets in the rank order of *rpoB>aroK>ppk>rpoC>ilvB*. *RpoB* was used as the cidality control. In-vitro and in-vivo studies feature *aroK* (encoding shikimate kinase) as an in-vivo mycobactericidal target suitable for anti-TB drug discovery. In-vitro to in-vivo cidality correlations suggested the low pH (R = 0.9856) in-vitro model as best predictor of in-vivo cidality; however, similar correlation studies in pathologically relevant (Kramnik) mice are warranted. In the acute infection phase for the high fidelity translation, the compound efficacy may also be evaluated in the low pH, in addition to the standard replication condition.

## Introduction

It is given that the new generation of drugs that will be used to manage MDR-, XDR-, and TDR-TB must result in cidality under in-vivo conditions [1]. However, the factors that govern in-vitro to the in-vivo translation of cidality are far from obvious. Mtb encounters complex physiological situations due to inflammatory immune pressures in the human host, starting with phagocytosis by the macrophages and ending in the same niche- the macrophages. The phagosome-lysosome fusion causes an acidic environment [2], and a strong chemistry of nitroxidative free radicals [3,4] produced by the macrophages in the granuloma; accompanied by gradual deficiency of nutrients: Carbon [5], Nitrogen [6], Oxygen [7], etc. However, some Mtb populations may be replicating logarithmically [8]. Thus, Mtb faces multiple milieus, in the host that makes its survival more complex and challenging [9]. Finally, a narrow window of “decision” between the infecting/ persisting pathogen and the adaptive/innate host immune response (immuno-competent/immuno-compromised), determines the judgement: disease or no disease [9]. Rest is the paradoxical hide-n-seek between the two, with their ambush (invasion and phagocytoses) and artillery (triggering or blocking various anti-inflammatory responses).

Conventionally, the drug discovery starts with the in-vitro screening of inhibitors. It is important to have the right assay condition/s for selecting potent molecules that must translate into in-vivo animal efficacy, the final proof of concept (POC). However, the standard in-vitro screens often do not adequately represent in-vivo physiologies. Therefore, one has to find an appropriate in-vitro model to predict efficacy, because every inhibitor cannot be validated through animal models. Usually, the failure of drugs to reach the clinic is attributed to two primary reasons: right efficacy and the right safety. It is economically and strategically prudent to fail at an early stage of inhibitor-screening rather than at a later stage of drug development.

In the present study, we tried to mimic the entire in-vivo relevant physiological milieu under in-vitro conditions. Our objective was to find out a robust link for in-vitro to in-vivo translation.

We used antisense-RNA (AS-RNA) silencing to inhibit the selected potential cidal targets from TB genome [10,11], including *rpoB-* the target of the tuberculocidal drug rifampicin; under all the simulated in-vitro conditions, as well as the in-vivo in the immunocompetent mice BALB/c. It was followed by a correlation of cidality from in-vitro screens with in-vivo cidality data. AS-RNA has a great potential in validating the therapeutically cidal *vs*. static targets for drug intervention in human diseases and selecting cidal anti-mycobacterials [8,11–14]. Here, we report the application of in-vivo AS-repression to demarcate the ideal assay conditions and tuberculocidal targets.

Our comprehensive AS-RNA silencing studies on the translation of target cidality from in-vitro to in-vivo revealed that *AroK* is the in-vivo validated target that culminated from the cidality SCORE. It emerged as an “in-vitro total” and “in-vivo” cidal target, whose inhibition is expected to be lethal to Mtb clinically. This study also raises the possibility of developing AS-RNA based therapeutics for treating TB patients in the long run. The low pH assay appears to be a critical in-vitro physiological condition that predicts the bactericidal potential of targets and correlates positively with in-vivo efficacy.

## Materials and Methods

### Bacterial strains, media, and antisense recombinants

Bacterial strains of *Escherichia coli* (MOS Blue cells {F’endA1 hsdR17 (rK2 mK+), supE44 thi-1 recA1 gyrA96 relA1 lac [F’ lacIqZDM15 proAB + Tn10 (TetR)]}, Amersham), *Mycobacterium smegmatis* mc^2^155, and *Mycobacterium tuberculosis* H37Rv ATCC 27294 were used for this study. We rationally selected five target genes for this study from the list of Sassetti’s classification on essential targets [11] i.e. *rpoB*, *rpoC*, *aroK*, *ilvB*, and *ppk*. The recombinants of these target genes for AS-RNA were generated by cloning full-length genes (sequences were taken from KEGG and Tuberculist databases) of these in the reverse/ antisense orientation into the vector pAZI9018b by replacing the *lacZ* gene [8].Three different independent transformations were performed for the target AS constructs. The AS recombinants of Mtb were selected from 7H10 agar plates supplemented with 50μg/ml Hygromycin (Hyg50) and were grown in 7H9 broth containing Hyg50. The O.D.600 nm was adjusted to 0.1, and the cells were induced at 10 μM IPTG. Transformants showed a slower growth rate, so wherever required; the suspensions were concentrated to match the required O.D. (O.D.600nm to 0.1, approximately 10^7^ cells/ml).

### Target selection for in-vivo validation

Though there are about 600 in-vitro essential genes in Mtb [11], we made a particular choice by selecting a few key target genes from different pathways: 1). Transcription: *RpoB* (Rv0667) and *rpoC*(Rv0668), encoding β and β’-subunits of RNA polymerase. *RpoB* is a well known clinically validated target, hence was also used as a bactericidal target control for this study. Its partner *rpoC* which is involved in the same process of transcription was selected to compare if it has an equal partnership and cidality potential. 2). Fundamental amino acid (aa) biosynthesis pathways: We chose two targets, *aroK*, and *ilvB*, one each from two amino acid biosynthesis pathways; aromatic (shikimate) and branched chain aa pathways. Both the targets *aroK* and *ilvB*, being in-vitro essential and have no human homologs, present a great opportunity towards the development of non-toxic drugs [15–18]. *AroK* (Rv2539c), from aromatic (shikimate) aa pathway, encodes the 5^th^ enzyme shikimate kinase, phosphorylating shikimic acid to shikimate-3-phosphate during chorismate biosynthesis. *IlvB* (*ilvB1*, Rv3003c)from branched-chain aa pathway, encodes Acetohydroxyacid synthase, (AHAS), the 1^st^and most important enzyme [19] out of the several genes (*ilvB1*, *ilvB2*, *ilvG*, and *ilvX*). Among all the genes in shikimate pathway, *aroK* is the only gene which is exclusive and in-vitro essential in Mtb. The essential amino acids can conditionally modulate bacterial growth [8,15,16], hence may act as regulatory tools to delineate their essentiality. Mtb exists in multiple milieu in-vivo [9], so, the targets of amino acid biosynthesis pathway were chosen as suitable tools for concept validation under various physiologies encountered in-vivo. In our earlier report [8], we have demonstrated in-vitro cidality of *ilvB* under replicating growth condition [8,12]. It is regulatable with the addition of physiological concentrations of ILV and P (Isoleucine, Leucine, Valine and Pantothenate) showing auxotrophy [20,21]. 3) The central energy metabolism: *Ppk* (Rv2984)or polyphosphate kinase plays an important regulatory role in the transition of the bacteria to the persistence phase under the growth-limiting conditions (phosphate depletion, amino acid starvation, or osmotic stress). It tries to accumulate polyP intracellularly, modulating several bacterial processes (protein synthesis, nucleotide balance, lipid metabolism, energy utility, and susceptibility to antibiotics)[12]. *Ppk* was chosen as an unvalidated target for this study because this target gene has not been characterised as per Sassetti’s list [11]. It is neither reported as essential (in-vitro or in-vivo) nor a non-essential target [11]. Earlier, we had established a partial validation of *ppk* as essential and cidal target under in-vitro replication condition only [12]. Hence, we chose *ppk* for this study as an un-validated target gene for further characterisation of its cidality under in-vivo condition.

Other than the clinically validated target from transcription subunit *rpoB*, rest of the targets *ppk*, *aroK*, *rpoC*, and *ilvB* are unvalidated targets since their in-vivo essentiality in Mtb is not reported yet. Therefore, in order to delineate in-vivo bactericidal potential of these targets in the present study, we used IPTG (Isopropyl β-D-1-thiogalactopyranoside) inducible AS-RNA silencing [8] as a gene-specific inhibitor for the chosen targets. It was used under multiple physiologies of granuloma simulated in-vitro (replicating conditions, and, under the stressful condition of nitrosative free radicals, acidic, hypoxic, or under starvation of C and N2) as well as under in-vivo conditions (experimental TB of mice).

### In-vitro cidality of Mtb targets under replicating conditions using survival kinetics after AS-RNA inhibition

The antisense effect was estimated in triplicate from 3 independent transformants for AS recombinants of *rpoB*, *rpoC*, and *aroK* in Mtb. The conditional AS-gene-silencing was induced by 10 or 100μM [8] IPTG under an in-vitro replicating growth condition. The survivors (from AS-silencing) were enumerated by plating on different generation times (Day 0, 1, 7, 14, 21, up to 63 days with a gap of 1 week), and the data was analysed using Prism software (Graph Pad Software, Inc., San Diego, Calif.). Appropriate controls were used. Two earlier established cidal targets (*ppk*, *ilvB*) for AS-repression under only the in-vitro ‘replicating growth conditions’[8,12]; were used as positive controls in this entire replication condition AS-silencing study. The clinically validated target, *rpoB*, was another cidal positive control throughout for all the validations. The negative controls for antisense repression were the WT = wild type Mtb; and the V = empty vector in Mtb (also the control for the recombinants). The genes *aroK*, *ilvB*, and *rpoC* were selected as un-validated target genes along with another uncharacterised target gene *ppk*.

### In-vitro cidality of Mtb targets under different physiologies

A total of seven different Mtb strains, i.e. five AS-recombinants (*rpoB*, *rpoC*, *aroK*, *ilvB*, *ppk*) and two control strains (WT and vector in Mtb) were taken for this study. We had to profile the survival kinetics of all the selected seven Mtb strains/ AS-recombinants, in triplicate, under six different assay conditions, (Platings/day = 7strainsX 3plicateX 6assaysX 5dil.sX 9time points) by plating 5 dilutions for cfu, up-to 35 days, under the constraints of bio-safety level-3 containment facility. A throughput method was ideal to conduct and compare all the experiments with replicates in parallel. Hence, a validated throughput SPOT-MBC assay [22] was used for this study because of its efficient way of testing a large number of samples/replicates from various in-vitro models, to be explored in parallel under BSL3 containment. Moreover, this relatively straightforward throughput method has been validated for different phenotypes of Mtb (AS-recombinants, vector controls in Mtb as well as WT, sensitive or resistant Mtb) as well; and had yielded cfu counts similar to conventional plating method [22]. The standard conventional plating method would have given an identical output; hence, it was prudent to use this efficient SPOT-MBC assay system. The *ppk* target was validated earlier [22] with SPOT-MBC and hence was taken as a positive control for this validation. The rest of the selected uncharacterised targets (*rpoB*, *rpoC*, *aroK*, *ilvB*) were validated in this study by conventional *vs*. SPOT-MBC methods in parallel under in-vitro replicating growth conditions, before using it for various physiological screens.

We further investigated the vulnerability of selected genes by checking cidality under all the in-vivo simulated multiple physiological conditions (the equivalent of macrophages and the lung granuloma) by performing survival kinetics in-vitro. All the strains were tested under six different physiological in-vitro assay conditions for investigations in parallel, in triplicate: replicating, hypoxia [7], nutrient starvation [5], low pH [2], nitric oxide stress [3], and nitrogen starvation [6]. The survivors were monitored by enumerating up to 35 days in a kinetic manner from all the screens in parallel using SPOT-MBC assay [22]. Since our previously reported *ppk* AS-repression (SPOT *vs*. conventional assay) experiments were performed only under proliferating assay conditions [12,22], and no other alternate physiological environments; we, therefore, investigated *ppk* AS-repression under all other the physiological conditions as well.

### In-vivo cidality of Mtb targets after AS-RNA repression

All the seven Mtb strains, including the controls and AS recombinants, were investigated for their growth or survival kinetics under in-vivo conditions in BALB/c mice, for confirmation of in-vitro cidality. This important in-vivo evaluation provided us with the proof of concept on cidality of targets under experimental TB in mice, the actual disease condition.

#### Ethics statement

The in-vivo study was carried out in strict accordance with the recommendations of the Institutional Animal Ethics Committee (IAEC), registered with the Committee for the Purpose of Control and Supervision (CPCSEA), Government of India (registration no. CPCSEA1999/5). All the protocols for animal experimentation and animal usage were reviewed and approved in advance by the IAEC. Carbon dioxide (CO_2_) was used for euthanasia.

#### Animals

Male BALB/c mice were purchased from RCC Hyderabad, India. Mice (6–8weeks) with an average body weight of 20–25 grams were randomly assigned to groups of three per cage and were allowed two weeks of acclimatisation before experimentation. The animals were housed under standard conditions in the facility with a 12 hr. day-night cycle. The infected mice were maintained in individually ventilated cages (Allentown Technologies, USA) in bio-safety level 3 (BSL-3) facilities. All the procedures including dosing of the infected mice were performed under strict BSL-3 bio-containment guidelines. Feed (the sterile commercial diet) and water were given *ad libitum*. We provided IPTG to mice in their drinking water at a 5mM concentration, as per the earlier established methods[23,24], for in-vivo target induction/repression. The animals were euthanized with CO_2_at the respective time points, and the lungs were sterically removed, homogenised and were plated for cfu enumeration of survivor Mtb bacilli.

#### Course of Infection (COI) studies in mouse-tuberculosis infection model

BALB/c mice were infected in the bio-safety level 3 (BSL3) facility, via inhalation in an aerosol infection chamber [25] with the suitable modification that delivered ~10^4^ bacilli/mouse lung, a higher dose of infection [26]. This method was standardised and validated in our laboratory [25,26]. The groups of mice (n = 3) were infected with different Mtb strains (WT and AS-recombinants of Mtb). High dose bacterial infection to lungs was delivered by increasing the strength of the bacterial inoculum (10^9^ cfu/ml) used for inhalation [26] that delivered 10^4^ cfu/lung, to allow evaluation of cfu reduction by capturing enough window following AS-repression. Instillation of 10^4^ cfu to lung/mouse was enumerated by harvesting and plating the lungs at day 3 post infection. The course of infection (COI) of all the strains was monitored on 3, 7, 14, 28, 42, and 56 days post infection by cfu enumeration.

Rifampicin was used at 30mg/kg bw as a reference drug control (for the WT Mtb) formulated in CMC (Carboxymethyl Cellulose) suspension [25]. Drug treatment with rifampicin started after 3 days following the establishment of acute infection. The drug was administered by oral gavage. AS-repression was induced with IPTG, given as 5mM in drinking water *ad libitum* beginning on day 3, based on the previously validated and established method [23,24]. The water bottles containing IPTG were replaced every 48 hrs. At every time point, the mice were euthanized with CO2, and the lungs were harvested and homogenized [25] in PBS containing 0.1% bovine gelatine and 0.1% triton-X100 using tissue grinders (W012576; Wheaton). Each suspension was serially diluted in 10-fold steps and plated on Middlebrook 7H11 agar supplemented with 10% albumin-dextrose-catalase. Plates were incubated at 37°C with 5% CO2 for 3 weeks, and colony forming unit (CFU) counts were enumerated on 3, 7, 14, 28, 42, and 56 days.

#### Transcriptome analysis of *in-vivo* antisense repression of target genes

Lung homogenates of AS-recombinants of Mtb along with the WT and vector control cultures were collected from different time points (day 3, 7, 14, 28, 42, 56), and pelleted. One ml of TrizolH was added to the cell pellets (from 2 ml homogenate/s) to stabilize and arrest the mRNA. These samples were flash frozen in dry ice and stored at -70°C until further processing. Wild-type (WT) *M*. *tuberculosis* culture control as well as the rifampicin-treated homogenates were also included. While processing, pellets were thawed on ice, cells were disrupted by bead beating using 0.1 mm diameter zirconium beads (Biospec), followed by a 5 min centrifugation at 14,000 g. Total RNA was isolated, and qRTPCR was performed using SYBR Green chemistry (Brilliant II SYBR® Green QPCR Master Mix) in the Mx3005P Stratagene system as reported [8,12] using respective forward and reverse primers (Table A in S1 File). The transcript levels were measured against *SigA* (Rv2703) as a validated house-keeping control gene, from our previous study of AS-repression [12], as well as taking clues from other reports including a detailed analysis by Manganelli et al. [27,28]. The fold difference was calculated against vector control using Delta-delta Ct method [29].

### Statistical Analysis

The data from in-vitro as well as in-vivo experimentation was analysed using Prism software (Graph Pad Software, Inc., San Diego, Calif.) for all the calculations for data. In-vitro data included survival plots as well as the correlation of cfu/ml from the SPOT-MBC *vs*. conventional plating method. The in-vivo data included the COI of various Mtb strains tested after AS-repression in-vivo. Two-way analysis of variance (ANOVA) with Bonferroni *post hoc* test correction was used to compare the net bacterial loads at each time point using Prism software (Graph Pad Software, Inc., San Diego, Calif.), to check the significance of AS repression for in-vivo cidality. The correlation between the outcomes from various in-vitro screens *vs*. in-vivo studies was analysed to narrow down to the best in-vitro screen, the predictor of in-vivo cidality.

## Results

### Antisense-silencing under in-vitro replicating condition

**Fig 1:**
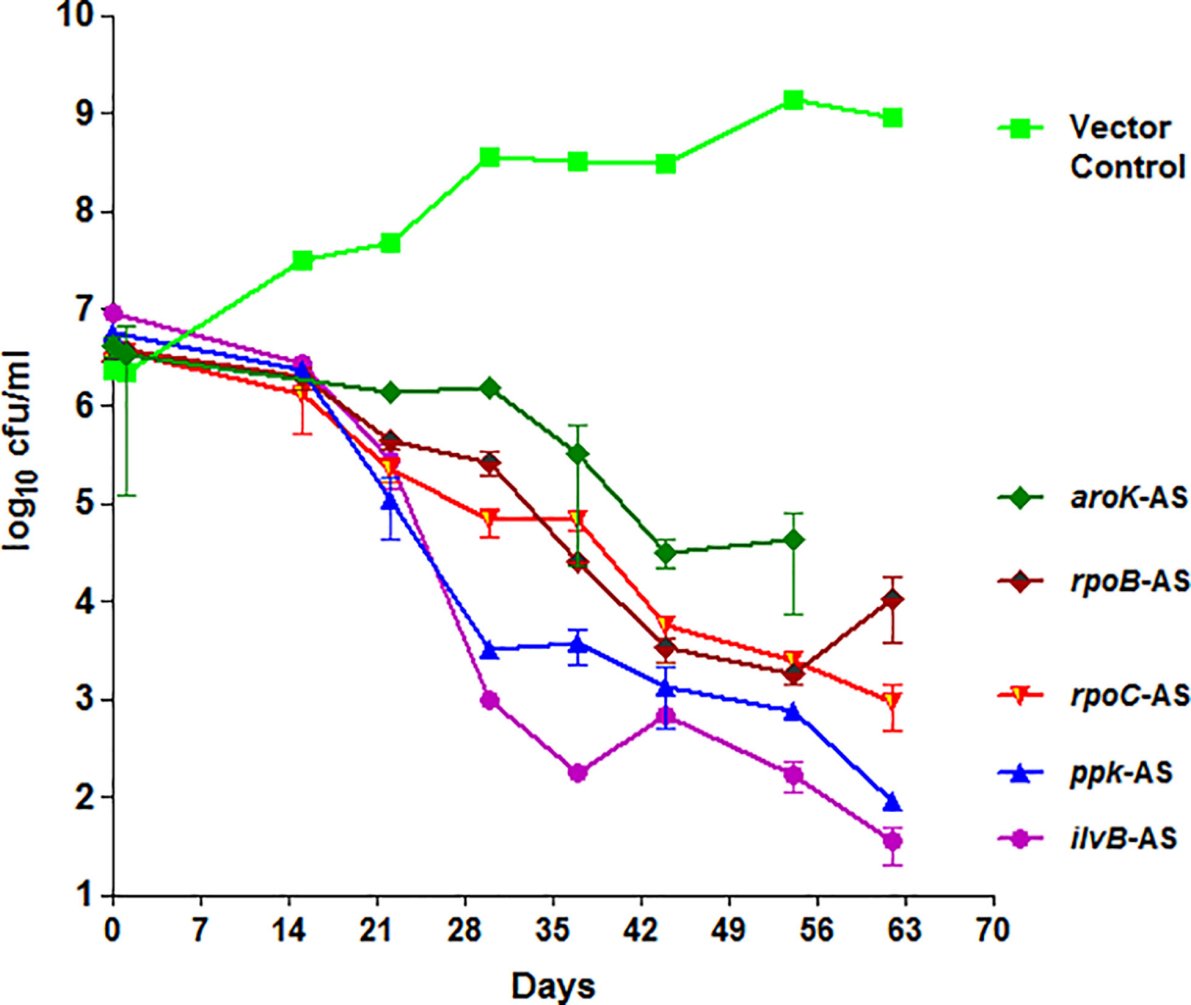
AS-silencing of Mtb targets under replicating in-vitro growth conditions. The survival kinetics of target AS-recombinants of Mtb enumerated up to 63-days = almost ~70 generations; are shown here as log_10_ cfu/ml *vs*. the vector control. Under replicating growth condition, *ilvB* demonstrated the maximum AS-repression among different magnitude of cidality in comparison to *ppk*. The order of cidality (log_10_ cfu/ml) was *ilvB* (5.4)*> ppk*(4.8)*> rpoC*(3.5)*> rpoB*(2.5)*> aroK*(2.2).

We selected the potential cidal target genes [11] *rpoB*, *rpoC*, *aroK*, *ppk*, *ilvB* of Mtb, and started with evaluating AS-gene-silencing [8] under in-vitro replicating growth condition. Complementation of the antisense (AS) and the sense (S) sequences de-stabilized the mRNA. Depending on the target vulnerability, translational blocking resulted in varying degrees of target specific AS-repression driven cidality of Mtb-recombinants as enumerated by log_10_ cfu/ml (Fig 1). The controls behaved as expected (vector as negative; and *rpoB*, *ppk*, and *ilvB* as the in-vitro-cidal positive controls). The known cidal ones were specifically cidal (*rpoB*). The order of cidality (≥2log_10_ cfu reduction) under replicating condition as observed on day-63 was: *ilvB* (5.4)*> ppk*(4.8)*> rpoC*(3.5)*> rpoB*(2.5)*> aroK*(2.2). On day-63, *ilvB* showed the maximum cidality (5.4 log_10_ cfu reduction) while in replicating condition, without hitting the maximum extent (Emax) yet.

### Throughput cfu enumeration assay for AS- silencing in-vitro

Validation data of all the AS-recombinants by the throughput “SPOT-MBC assay” *vs*. conventional [22] plating for cfu enumeration (S1 Fig) demonstrated an overlapping data with a strong positive correlation (Pearson’s): *rpoB* (R^2^ = 0.5857), *rpoC* (R^2^ = 0.7770), and *aroK*(R^2^ = 0.9863) *vs*. vector controls. However, in the case of *ilvB*, we found a variation in the Emax (*SPOT = 3*.*3 log*_*10*_ *and conventional = 5*.*2 log*_*10*_ *reduction*), probably, the smaller surface area of the agar media in SPOT-assay, allowed the colonies to grow in proximity. It enabled socializing through the exchange of the required amino-acids between the healthy and the sick or nearly dead bacilli, helping them to rejuvenate. Overall, the SPOT-MBC assay was aptly and significantly suitable (EEE) [22] for kinetic profiling of Mtb AS-recombinants and controls under multiple tests. Henceforth, spot assay was used for cfu enumeration for all the in-vitro screens.

### AS-silencing under physiological stress conditions in-vitro

**Fig 2:**
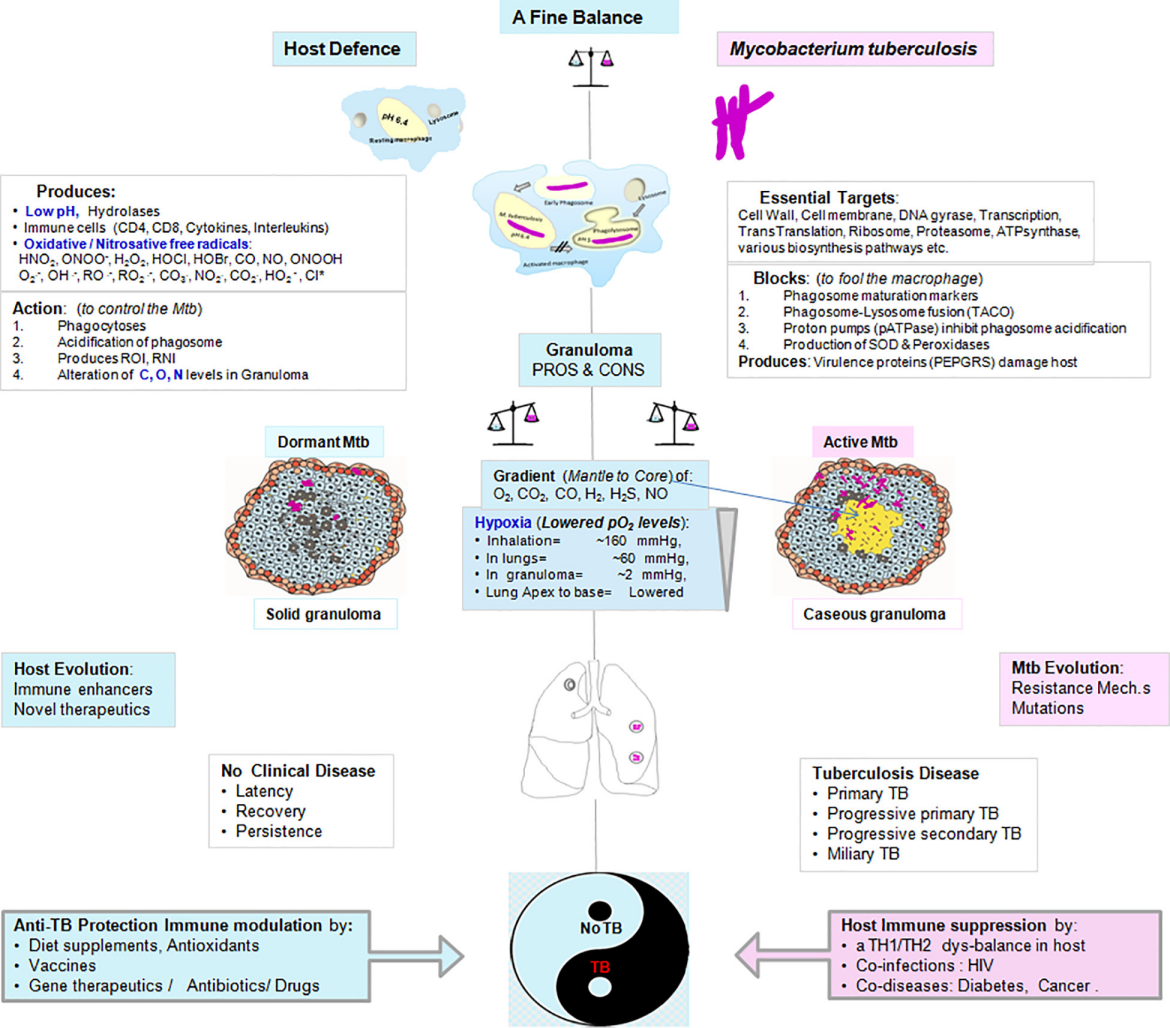
TB or No TB: A delicate balance between host-pathogen interactions. There are various hostile or stringent conditions encountered by Mtb in-vivo in the host. Mtb enters the host via inhalation. The first encounter in-vivo is with the immune cells: the macrophages which phagocytose and attack Mtb with their ammunition like low pH, hydrolases, free-radicals, etc. This first encounter is unanimously responded by all the hosts irrespective of their immune status. Beyond this point, the countering of the pathogen is host population specific. The outcome as TB or no-TB is a delicate balance and is outcome result of the battles between the pathogen and the immuno-competence of the host to outsmart. Further, it progresses into a larger immune structure: the granuloma. During the process, the other stresses in the granuloma, especially in the caseating/ necrotizing lesions are the gradually decreasing levels of O_2_, N, C, etc. representing hypoxia, the arrest of various biosynthesis processes and poor nutrition nearly close to starvation. If the host wins, the pathogen is contained in the solid granuloma, which may gradually heal with time. But in case the pathogen Mtb overpowers the immune pressure, the granuloma progresses as a caseating and necrotising granuloma. In this case Mtb bacilli multiply to finally break open from granuloma, and disseminate to other organs of the body or come out into the bronchi and get coughed out to infect other hosts. We attempted to simulate some of these stress conditions in the form of various in-vitro screens to identify the ideal in-vitro screen/ condition.

**Fig 3:**
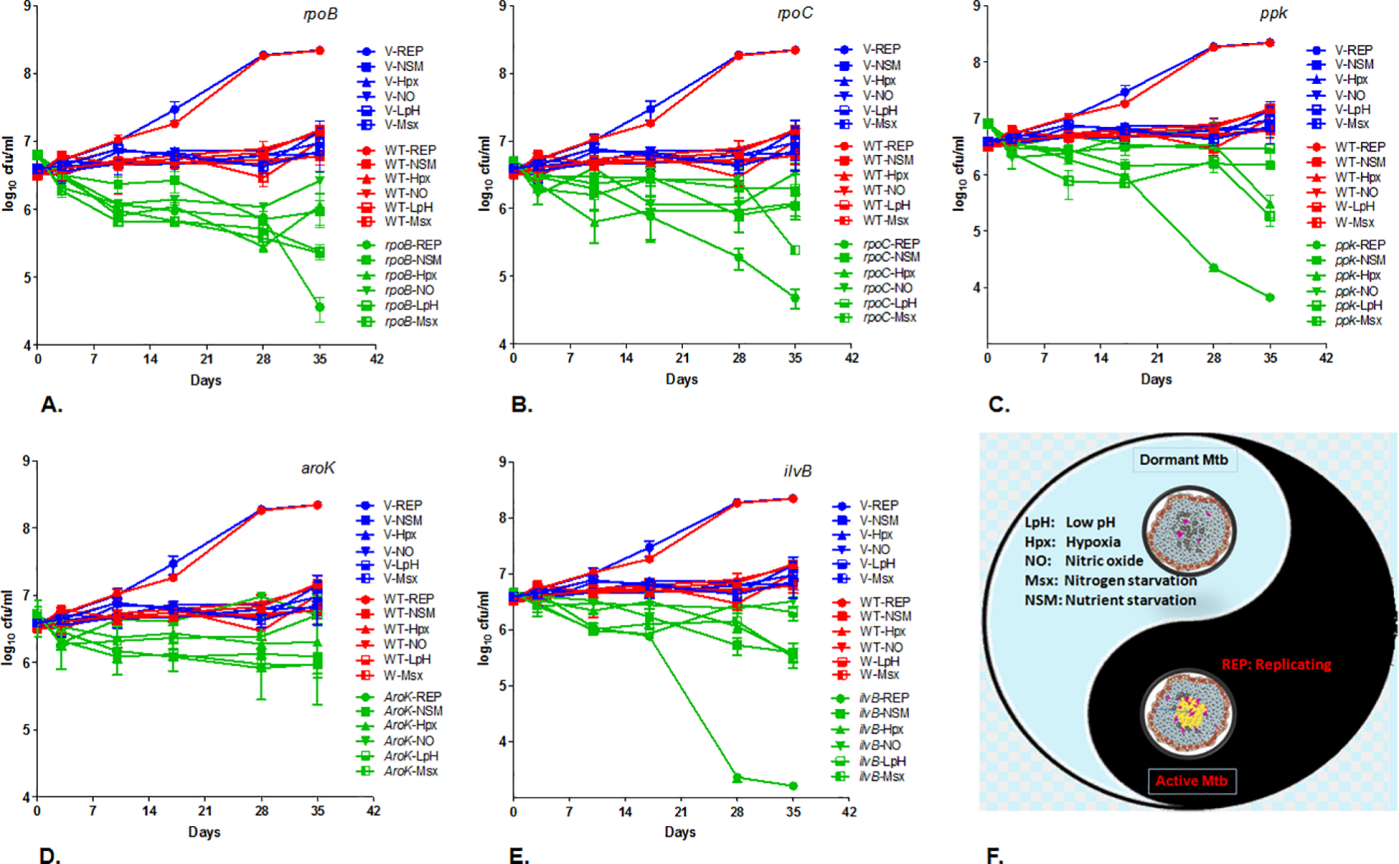
Survival kinetics of AS-silenced Mtb under different in-vitro physiologies. Survival kinetics of AS-recombinants: (A) *rpoB*, (B) *rpoC*, (C) *ppk*, (D) *aroK*, and (E) *ilvB*; under granuloma simulated (F), replicating and non-replicating (dormant) in-vitro growth conditions, showed target vulnerability specific cidality. Various conditions tested: Replicating (REP) growth condition as solid circles, Nutrient starvation (NSM) as solid square, Hypoxia (Hpx) as solid vertical triangle, Nitric oxide (NO) model as solid inverted triangle, Low pH (LpH) as bottom solid square, Nitrogen depletion (Msx) condition as the left solid square. The graph represents plots of log_10_ cfu/ml *vs*. no. of days, studied up to 35 days. V and WT are plotted with all the genes as controls for a comparison. All the symbols have been kept uniform for the respective assay conditions throughout. WT is RED; Vector is BLUE, and the gene-specific AS-recombinants of Mtb are in GREEN colour. The error bars (SEM) from independent triplicates represent the robustness of data. The data for WT and V are common in all the graphs, for an easy comparison with the green ones- the gene specific. Although Mtb can withstand and emerge successfully from various physiological pressures encountered in-host; a target is superior if, it is bactericidal under all or most of those physiological constraints upon specific inhibition. The targets were significantly (P<0.001) silenced with different cidality magnitudes: *ppk* (7.5)>*ilvB* (7.3)>*rpoB* (7.1)>*rpoC* (5.2)>*aroK* (3.2).

AS-driven survival kinetics, under various alternate physiological in-vitro conditions (Fig 2), was a yardstick to understand the overall importance (cidality) of respective targets. As expected, the WT and V controls exhibited condition-specific, normal growth under all the physiologies. Since *ppk* was reported cidal under proliferating assay conditions [13,22]; in this study, under different physiological conditions, *ppk* exhibited the maximum total AS-repression among the tested. Whereas, under the replication condition alone, *ilvB* had demonstrated maximum cidality. The other targets were also significantly (P<0.001) silenced with different cidality magnitudes: *ppk*(7.5)*>ilvB*(7.3)*>rpoB*(7.1)*>rpoC*(5.2)*>aroK*(3.2) (Fig 3). The stringent conditions where most of the targets were cidal, showed a rank-order for the starvation of N>C>O>pH. Surprisingly, the genes *rpoB*, *rpoC*, *aroK*, and *ppk* were significantly repressed upon the exposure to acid. However, starvation data revealed that Nitrogen depletion (Msx) accelerated “silencing of most of the targets in-vitro” followed by nitric oxide (NO) and low pH (LpH) pressures as compared to the replicating (REP) growth conditions.

### Cidality SCORE

**Fig 4:**
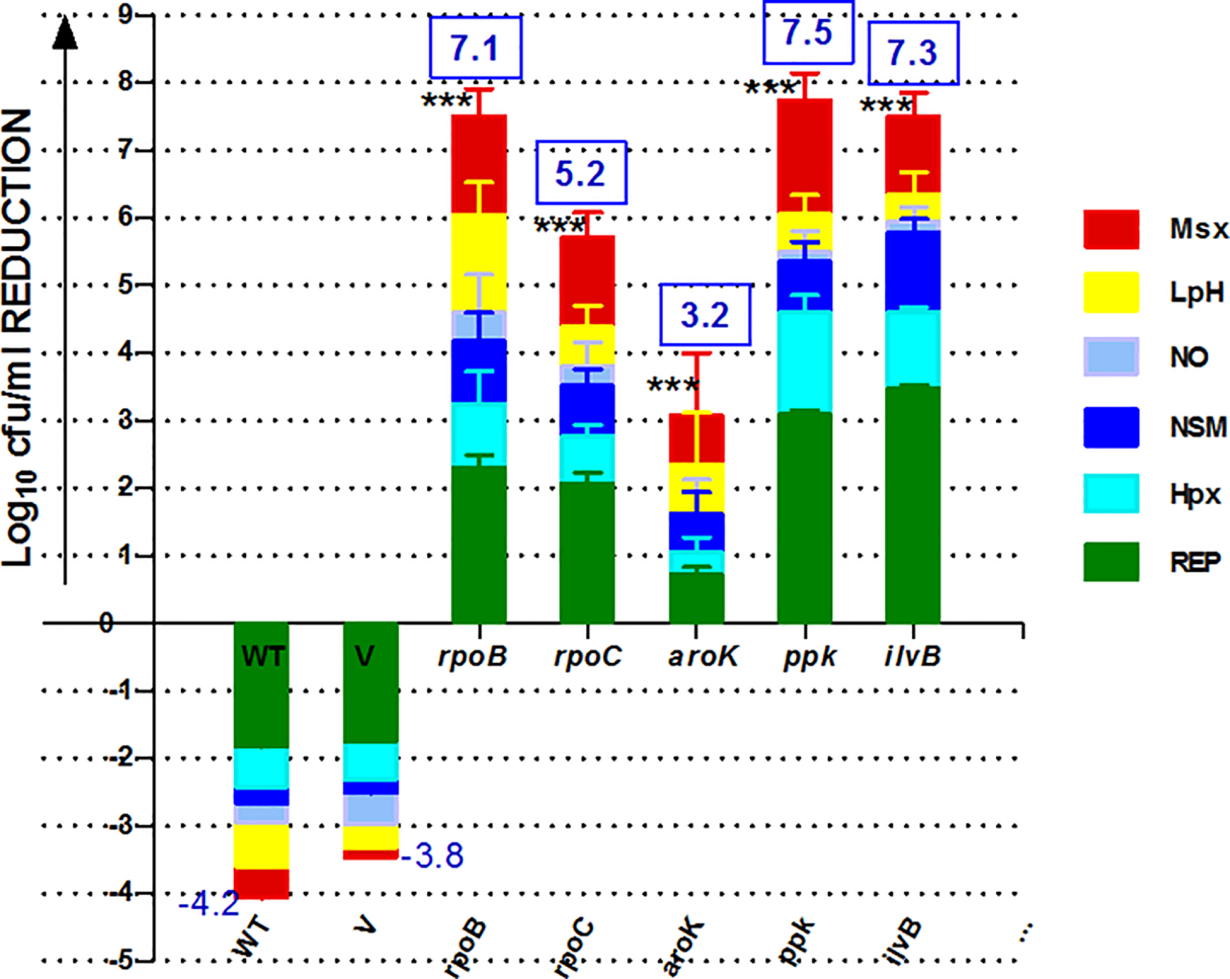
Cidality SCORE of Mtb targets by in-vitro AS-silencing. The normal or stringent physiological conditions used are: Msx = Nitrogen depletion, LpH = low pH, NO2 = Nitric oxide, NSM = Nutrient Starvation Model, Hpx = Hypoxia, REP = logarithmically replicating condition. The graph depicts the net compounded effect, the Cidality SCORE, of respective genes under various physiological conditions (a total of) as the inhibition on the upper scale, and growth on the lower scale. It represents the behaviour of the respective target under a diseased situation. WT = Wild-type Mtb strain, V = WT strain of Mtb containing the blank vector. The rest are all the gene-specific recombinants of Mtb. The blue colour boxes show the cidality SCORE representing the overall cidality potential of a target based on the AS-RNA gene silencing magnitudes as *ppk*(7.5)>*ilvB*(7.3)>*rpoB*(7.1)>*rpoC*(5.2)>*pyrH*(3.6)>*aroK*(3.2). Statistically significant (***), the error bars (SEM) represent the robustness of data from the triplicates.

TB is a complex disease where Mtb can efficiently demonstrate multiple dimensions of pathogenesis under paradoxical milieus of the host. We asked the question if the total compounded sum of all these stringent pressures on a target would show a potential link between in-vivo persistence; as it would behave under multiple milieus in a diseased situation? Therefore, we calculated a ‘compounded potential’ of the chosen targets. The SCOREs were assigned based on the extent of AS-repression of targets, representing the net cidality of a target from all the replicating/ non-replicating screens (Table B in S1 File, Fig 4) whether negative (growth) or positive (kill). The cidality SCORE graphs were plotted (Fig 4), demonstrating the total potential (vulnerability) in the following order of target cidality (log_10_ cfu reduction): *ppk*(7.5)>*ilvB*(7.3)>*rpoB*(7.1)>*rpoC*(5.2)>*aroK*(3.2). The resulting numeral indicated the “cidality SCORE” and a measure of cidality potential of the respective targets. The entire data was statistically significant (*Dunnett’s multiple comparisons*) *vs*. the controls. However, in the case of *ilvB*, an attenuation of the target was observed due to the revival of additional colonies upon extended incubation (up to 45 days).

The AS-repression under regular and stringent growth demonstrated that the in-vitro cidal targets showed maximum SCOREs up to 7.5 (log_10_cfu reduction), compared to the controls (Fig 4). This outcome of the in-vitro alternate physiological conditions established specifically few targets (*rpoB*, *aroK*, *ilvB*) cidal under all the conditions (Fig 4). Our next objective step was to validate cidality of these targets under the in-vivo experimental model of tuberculosis.

### In-vivo AS-silencing of in-vitro cidal targets in mice

Validation of the SCORE based in-vitro cidality was confirmed in-vivo by infecting BALB/c mice with the respective Mtb strains (AS-recombinants and WT and vector controls) via an aerosol route [25,26]. In-vivo studies were approved by IAEC (CPCSEA), as mentioned in the ethics statement. The in-vivo AS-silencing was achieved by providing IPTG (5mM) to mice in drinking water [23,24], a non-invasive technique of IPTG delivery in drinking water; thereby minimising the handling of infected mice in the bio-containment set-up.

#### In-vivo cidality of the targets on 28^th^ day

**Fig 5:**
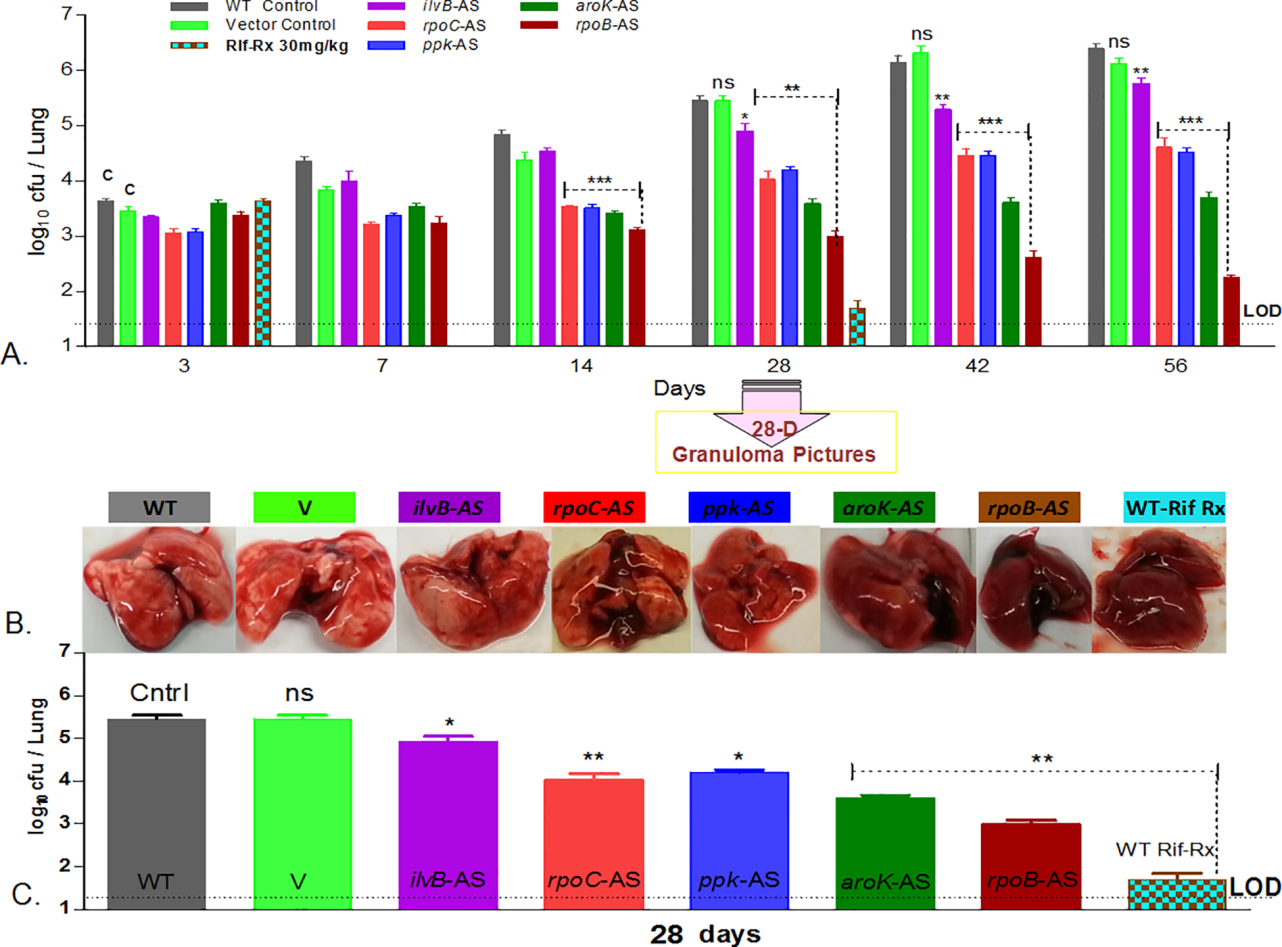
Cidality of Mtb targets by AS-silencing in lung infection in mice. (A). Overall survival kinetics of Mtb AS-recombinants and the WT and vector controls in the lungs of mice (n = 3) on the days-3, 7, 14, 28, 42, 56. The control strains showed expected course of infection in the lungs; there was no difference in both these strains (WT and vector, ns, P = 0.3105, two-way ANOVA). The treatment control (Rifampicin treatment of WT Mtb) showed expected cidality pattern of ~3.8 log_10_ cfu reduction, represented by the turquoise and brown chequered bars on day-3 and day-28. Target *ilvB* (however, significantly different from control, *P = 0.0402, two-way ANOVA) was non-cidal in-vivo. The graph is a plot of log_10_ cfu/lung of mice *vs*. number of days. The cidality emerged in the rank order of *rpoB>aroK>ppk>rpoC*. Data was statistically significant (**P = 0.0086 to *P = 0.0402, two-way ANOVA) from 14 day onwards. (B). Lung pictures on the day 28 (4^th^week), visually demonstrate in-vivo cidality by a clearing of infection in the lungs with the healing of granuloma due to the killing of the respective AS-recombinant of Mtb in the order of *rpoB>aroK>rpoC>ppk*; correlating with the cfu data outcome (panel **C**). The maximum healing was visible in the rifampicin treated lungs, correlating with the cidality shown in the cfu histogram. The control strains (WT and V) demonstrated the fully formed visible granulomas in the lungs. (C). Final histograms of AS-based cidality on Day-56 (8^th^ week) graphs. A statistically significant robust data with error bars (SEM) from triplicates (n = 3), shows the cidality pattern in the order of *rpoB*(3.9)> *aroK*(2.4)*> ppk*(1.6)*> rpoC*(1.59)>*ilvB*(0.36).

The first visible and quantitative in-vivo trends of cidality appeared in the lungs after four weeks post-infection. The control Mtb strains grew as expected, with fully developed visible granulomas in the lungs (Fig 5), whereas the AS-recombinants showed reduced growth on the 28^th^ day, due to target specific AS-repression and cidality. *RpoB* showed the maximum cidality in-vivo (3.9 log_10_ reduction), despite only 2.5 log_10_cfu reduction in the replicating in-vitro model. The culture plates were incubated up to 45 days, and the colony characteristics were observed. None of the AS-recombinants, except *ilvB*, showed any signs of re-growth or delayed appearance of additional colonies on the media plates. The colony counts remained constant upon further incubation; suggesting cidality due to AS-repression in *ppk*, *rpoB*, *rpoC*, and *aroK*. On the contrary, in the case of *ilvB*, the appearance of additional colonies on extended incubation point towards attenuation rather than cidality. *IlvB* showed a negligible cidality (0.36 log_10_cfu reduction) under the in-vivo condition on day 28 and exhibited granuloma formation similar to the control strains. Targets like *ppk*, *rpoC* and *aroK* were repressed by their respective AS-counterparts and showed significant (two-way ANOVA,**P = 0.0086 to *P = 0.0402), but varying levels of log_10_ cfu reduction: *rpoB*(2.5)> *aroK*(1.9)*> ppk*(1.2)*> rpoC*(1.5). The in-vivo model was validated by including a positive treatment control; using the rifampicin treatment (30mg/kg) of WT Mtb that demonstrated ~3.8 log_10_cfu reduction. This drug treatment control was a non-AS-RNA based inhibition control.

#### In-vivo cidality of targets on 56^th^ day

The bacterial reduction in the lungs clearly delineated cidal targets from the static ones. The rank order of log_10_cfu reduction magnitude was: *rpoB*(3.9)> *aroK*(2.4)*> ppk*(1.6)*> rpoC*(1.59); significantly different (***P = <0.0001 to 0.0004) when compared to the WT. The vector control Mtb demonstrated no difference from the WT strain (ns, P = 0.6283) (Fig 5). In contrast, *ilvB* (**P = 0.0012, two-way ANOVA) behaved as a static-to no-effect target under the in-vivo conditions. It demonstrated a marginal cfu reduction (0.36 log_10_ cfu reduction) *vs*. the control Mtb. The treatment (IPTG and Rifampicin) was initiated 3-day post-infection (10^4^ cfu/Lung), to substantiate and compare the killing kinetics of AS induction *vs*. the drug treatment. It was “more reflective of treating individuals who are recently infected” and not the chronic TB infection.

#### Pharmacokinetics (PK) of AS-RNA

**Fig 6:**
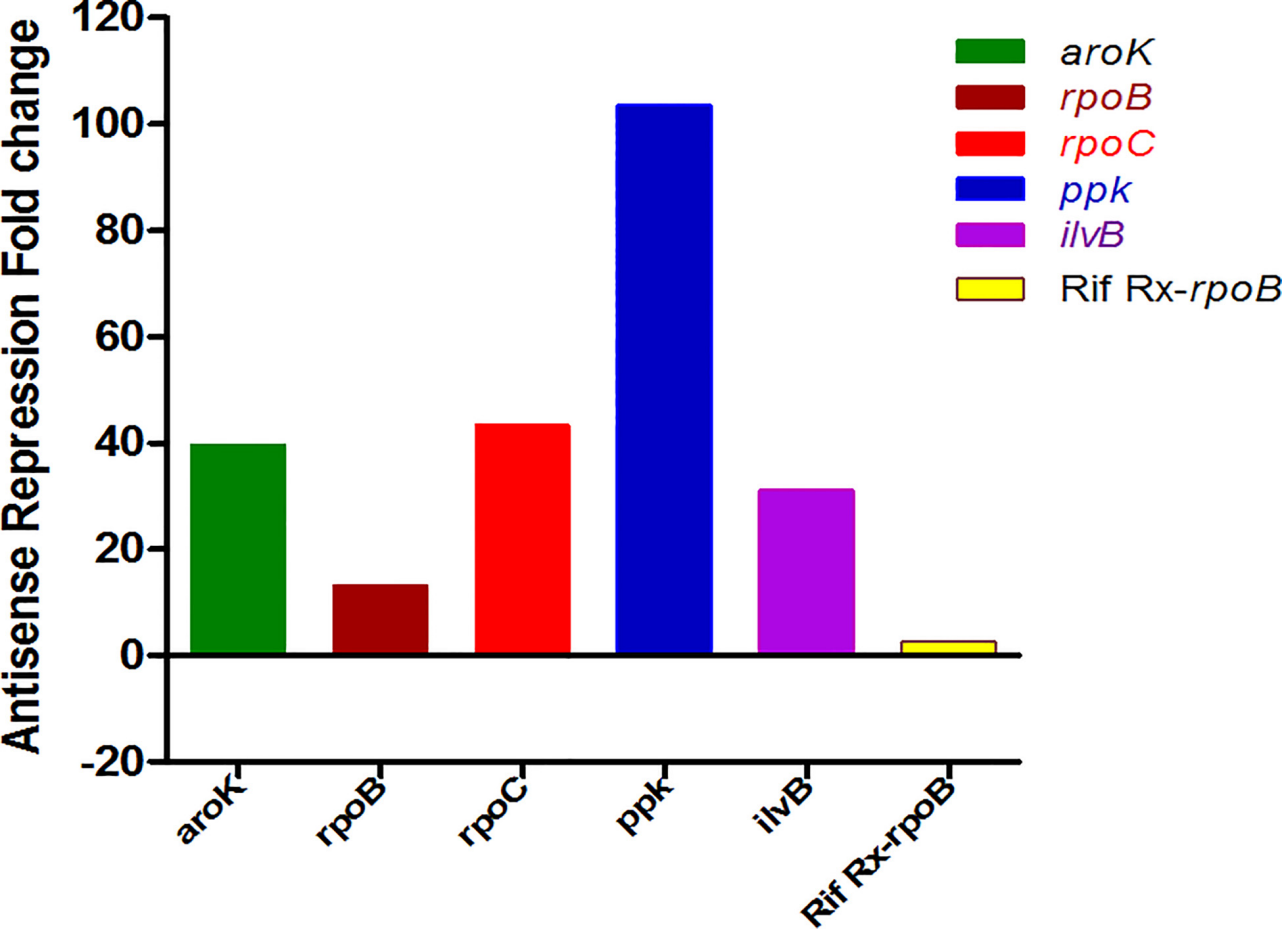
Maximum fold target repression during the course of infection. The data validates IPTG-inducible in-vivo AS-repression of Mtb targets. The net target transcript levels, as evaluated by RTPCR of Mtb from lung homogenates; showed a variable -fold down regulation (13- to 103-fold), during the entire course of in-vivo studies. The maximum fold repression of targets equated that the in-vivo transcript translation into cidality is target-vulnerability-dependent. Target *rpoB* translated into maximum cidality of 3.9 log_10_ cfu reduction with mere 13-fold transcript level repression; whereas, in the case of *ppk*, only 1.3 log_10_ cfu reduction could be achieved in-vivo despite a maximum of 103-fold transcript repression (as in Table C in S1 File).

We evaluated the levels of AS-RNA generated by the Mtb-recombinants (during the span of infection in mice), indirectly, by measuring the net fold target repression, representing bio-availability of AS-RNA. It was estimated by RTPCR (Table C in S1 File, Fig 6) of transcriptome from Mtb strains recovered from the infected lung homogenates. Each target was repressed, though with different magnitudes (13- to 103-fold), proving that Mtb AS-RNA had no PK issues. Although the order of cidality was *rpoB>aroK>ppk>rpoC>ilvB*; the transcript down-regulation rank order was: *ppk>rpoC>aroK>ilvB>rpoB* (Fig 6). The Rifampicin treated WT Mtb transcript showed a mere ~3-fold down-regulation of the target gene (*rpoB*, maximum 2.6 fold) in-vivo, deficient enough to show cidality of 3.8 log_10_ cfu reduction. Maximum cidality demonstrated by *rpoB* had the minimum fold transcript down-regulation, hence vulnerable. The maximum transcript was down-regulated in *ppk* that showed the least cidality. The safety and bioavailability of IPTG are reported earlier [23].

### Correlation of in-vitro score *vs*. the in-vivo cidality

**Fig 7:**
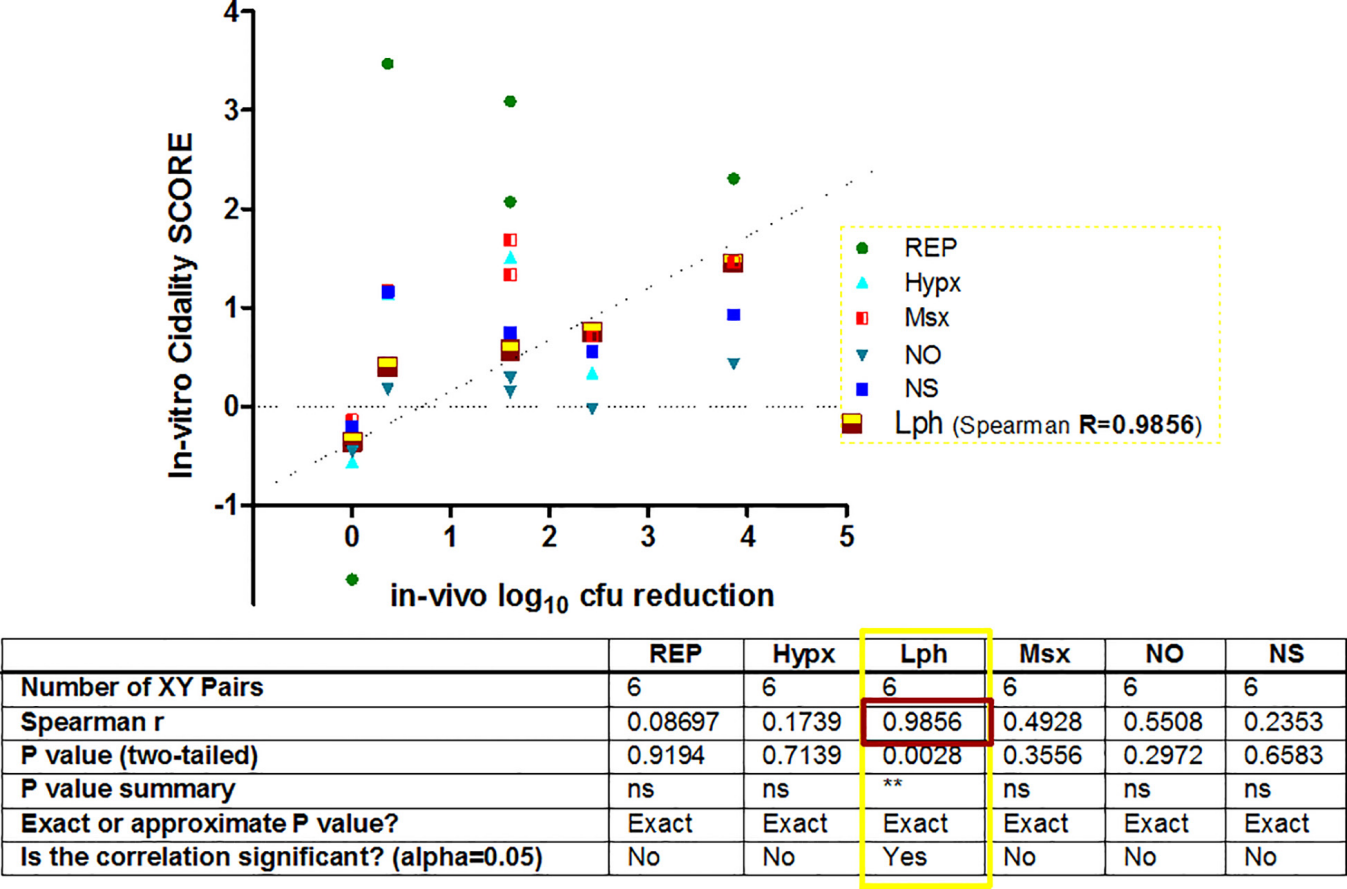
Correlation of Cidality under physiology of in-vitro *vs*. in-vivo. The low pH condition appears to be the ideal screening system (R = 0.9856) showing linearity during a correlation of different in-vitro screens of alternate stress responses *versus* the in-vivo outcome for selection of cidal inhibitors. REP = Replicating growth condition, Hpx = Hypoxia, Msx = Nitrogen depletion using L-methionine sulphoxide, NO = Nitric oxide, NS = Nutrient starvation of Carbon, and LpH = low pH condition. A statistically positive correlation (Spearman R = 0.9856) was observed between the in-vitro Low pH condition and the in-vivo outcome. The counts of in-vitro cidality SCORE are available in Table B in S1 File.

The in-vitro replicating/non-replicating data had a varied cidality/gene repression response under in-vivo conditions with different magnitudes. Most of the ‘in-vitro cidal’ targets (4 out of 5; 80%) demonstrated cidality under in-vivo condition. Cidality data from all the in-vitro assay conditions *vs*. the in-vivo efficacy data, showed a significant correlation (Spearman R = 0.9856) with the low pH in-vitro assay condition (Fig 7), establishing that the “low pH in-vitro screen” optimally simulated the in-vivo disease condition to select in-vivo cidal inhibitors.

### MIC of anti-TB drugs under low pH

Most of the frontline drugs had MIC on pH adapted Mtb cultures under low pH (6.2) condition (Table D in S1 File) except isoniazid.

## Discussion

The slow-growing intracellular Mtb remains focused on capturing the fortress of lungs. Mtb strategically evolves new sets of artillery (combination of genes) during the phases of replication and persistence. It implies that the conditionally expressed targets required in the in-vivo phase during the disease might be the relevant ones under all the host-physiologies to achieve cidality. It prompted us to identify the targets that are bactericidal under all in-vivo physiologies, like *rpoB*, the target of rifampicin.

In this study, we investigated various in-vivo simulated physiologies of the granuloma-like environment, under in-vitro conditions. Using AS-RNA-silencing as a cidality tool (genetic inhibitor), we examined five rationally selected targets cidal under all the in-vitro screens and correlated the outcome from in-vitro versus the in-vivo data. Our data demonstrated *rpoB* and *aroK* as the best mycobactericidal targets under all the physiologically relevant conditions. *RpoB* was used as a clinically proven cidal target control.

*AroK*(encoding shikimate kinase) emerged as an in-vivo validated Mtb-cidal and vulnerable target under all the physiologically relevant conditions, suggesting its potential for TB-drug discovery. Shikimate pathway has been previously reported as in-vivo essential only for *aroA* and *aroC* in the ESKAPE pathogen, *Acinetobacter baumannii* [30]. Few pathogens like *E*. *coli*, *Salmonella typhimurium*, and *Yersinia pestis* have one extra shikimate kinase gene isoform (*aroL*), which is catalytically superior, better regulated and plays a pivotal role [30–32]. On the contrary, no clarity on the physiological function of *aroK* suggests its non-essentiality in these pathogens [33]. On the contrary, in *M*. *tuberculosis* shikimate kinase is unique and exclusive enzyme essential for its survival; as is the finding reported in another intracellular pathogen *Helicobacter pylori* [15,16,34]. It presents an excellent opportunity for exploring *aroK* target for TB drug discovery. The in-vivo essentiality of *aroK* in Mtb has not been reported yet. However, in the case of *Acinetobacter baumannii*, a very recent report has confirmed aroK as an in-vivo essential enzyme [30,32].

In the present study, Mtb *AroK* performed consistently well across all the simulated physiologies in-vitro (cidality SCORE = 3.2) as well asunder in-vivo condition (2.4 log_10_cfu reduction;39.7-fold transcript repression in-vivo, Figs 1 and 3–5) despite a comparatively lower in-vitro cidality SCORE, thus, delineating its cidality. Blocking *aroK* target kills Mtb under in-vivo, clearly indicating nearly zero availability of amino acids (tryptophan, tyrosine, and phenylalanine) in the in-vivo milieu. However, whether it will translate into a therapeutically valid target for clinical usage is worth further investigations.

Our studies confirmed *rpoB* as the most vulnerable target with a mere 13-fold transcript repression in-vivo (Table C in S1 File) translating into an excellent in-vivo cidality (3.9 log_10_ cfu reduction). Emerging from a good in-vitro cidality SCORE (7.1, Fig 4) with cidality under low pH, it established that inhibiting *rpoB* with AS-therapeutics (unique MOA of blocking mRNA specifically) is equipotent to rifampicin treatment, validating our experimental approach. However, it’s another RNAP partner, the *rpoC*, demonstrated a lower “cidality SCORE” (and a negligible cidality in low pH) compared to *rpoB* (5.2*vs*. 7.1, Fig 4), though marginally better cidality under replicating condition than *rpoB* (Fig 1). Despite a high (43-fold) in-vivo transcript repression for *rpoC*, the cidality SCORE could, in fact, delineate *rpoC* as just a marginally in-vivo bactericidal target (1.6 log_10_ cfu reduction) as compared to *rpoB* (Table C in S1 File, Fig 5).

Targets, *ilvB*, and *ppk* were “highly in-vitro bactericidal” (Figs 1 and 4), but comparatively less cidal targets under in-vivo condition (Fig 5); despite their best repressed in-vivo transcript levels (*ilvB* = 31.1-fold, ppk = 103.6-fold; Table C in S1 File, Fig 6). One of the best in-vitro-cidal targets, *ilvB*, demonstrated negligible in-vivo cidality (0.36 log_10_ cfu reduction, Fig 5). This outcome is attributable to its auxotrophic nature [8,35] and suggests compensation of the effect by the availability of trace amino acids (isoleucine, leucine, valine, and pantothenate) under in-vivo milieu. It is hard to delineate attenuation and cidality, but probably *ilvB* flaunted target attenuation under the in-vitro condition as well, because of the revival of additional colonies upon extended incubation. The presence of trace amino acids (ILVP) nullified its cidality potential [8] leading to attenuation. Other targets studied (*ppk*, *rpoB*, *rpoC*, *aroK*), did not demonstrate this phenomenon, suggesting their bactericidal nature.

Next best in-vitro cidal target *ppk* (Figs 1 and 4), was marginally in-vivo-cidal (1.6 log_10_ cfu reduction, Fig 5) despite its maximum in-vivo transcript repression (103.6-fold; Table C in S1 File, Fig 6). Since *ppk* is proven to be a stationary phase specific target [12], a long-term infection model in mice may be required to demonstrate its cidality. The in-vivo model used for the present study was a hybrid model encompassing from acute to chronic infection states. We intentionally infected mice with high dose (10^4^ cfu/lung) to facilitate a measurable window for cfu enumeration, whether the bacterial numbers increase (up to 10^8^ cfu/lung) or reduce. To begin with, this mouse infection model is an acute model harbouring replicating bacilli. But with time (beyond four weeks) bacterial growth of WT control and the recombinant strains slows down, it acquires more of a nonreplicating state in-vivo while the AS effect is still on. Hence, the later part of the course of infection represents the AS-effect on a chronic disease. Since AS-repression based cidality was observed beyond 4 weeks as well (Fig 5), it suggested a possibility of killing Mtb even during the chronic phase. However, AS-based cidality studies in a chronic mouse infection model are warranted. Upon a continuous expression of AS-RNA under IPTG induction, the respective targets were being silenced during the course of infection in mice from acute to chronic; hence, demonstrating a stage-specific cidality effect, if the target was essential under the respective conditions.

Interestingly during 4–8 weeks period (chronic stage of infection) in particular, a further growth of *ppk* recombinant was significantly inhibited (ns, P = 0.1080, one-way ANOVA, Dunnett’s multiple comparisons) by the antisense. Whether the bacterial count will reduce further beyond 8-weeks, needs to be determined. However, we need to delayer it separately, with careful investigations in a long term course-of-infection model beyond 8-weeks.

The cidality SCORE correlated well with the in-vivo data outcome in 4 out of 5 targets (80%) confirming in-vivo-cidality. *AroK* target, which showed a moderate in-vitro cidality under replicating condition, would have been missed out if had not been checked under alternate physiological conditions as well. It outlines the importance of cidality-SCORE. A consistent performance of *aroK* under all the physiological conditions tested demonstrated a cidality SCORE of 3.2, and revealed its cidality potential; and hence, selected for in-vivo validation. It emerged as a bactericidal target under in-vivo condition also, probably because it was cidal under all the physiologies studied. *RpoB* was far more superior to *rpoC*. The cidality-SCORE approach was a worthwhile investigation, implying that a target is preferred if it is cidal under all the physiologies encountered in the host. It was apparently confirmed by *rpoB* and *aroK*, but not by *rpoC*. The cidality SCORE could intricately delineate the in-vivo bactericidal potential of *rpoB* and *rpoC* targets. The only target which did not show in-vitro to in-vivo correlation was *ilvB*, because of other issues like auxotrophy and attenuation observed in this study. Overall, the in-vitro cidality SCORE could predict the in-vivo cidality of the targets, irrespective of their magnitudes. Since frontloading of all alternate physiological screens in parallel may not be feasible; we questioned which screen out of the six conditions tested is the best in-vivo cidality predictor?

Antimycobacterials are tested under the replicating growth conditions in 7H9 media [8,36,37], and confer limited translation of in-vitro cidality into the in-vivo efficacy, thus resulting in high attrition rate in drug discovery. This physiology of ‘in-vitro replication’ growth condition does not represent the ‘actual microenvironment encountered under host immune pressures’ to a large extent, thus overlooking the selection of potential in-vivo cidal compounds. As a consequence, inappropriate compounds get selected. During the paradoxical intra-macrophagic phase, the acidification renders Mtb almost inactive, even before it understands the in-vivo milieu to adapt in [38]. The activation-specific pH of macrophage (~6.2), further drops upon phagosome-lysosomal (P-L) fusion to ~4.5 within 15 to 60 minutes under various immune pressures [39]. Despite this acidified external milieu of macrophage, the internal pH of Mtb (~7) remains neutral [40]. In order to refrain from the P-L fusion (Fig 2), and to retain itself within the macrophage to progress further, Mtb invariably fools the system by several mechanisms of interference by inducing virulence proteins and efficiently expressing macrophage-specific survival genes [39,41]. The stage-specific transcriptome levels in macrophage [42] fluctuate with the physiological transitions of replication and death rate (Table C in S1 File) in the host. The macrophage attack on Mtb is the 1st battle to be won by the host (Fig 2) representing the most significant universal host-pathogen interaction. It highlights the potential link of low pH to the intraphagosomal survival of Mtb [42–45], urging researchers to simulate this ‘in-host’ stage under a single in-vitro screen to select bactericidal agents [43,44]. We conducted comprehensive studies on in-vivo simulated microenvironments under in-vitro conditions and their correlation versus in-vivo cidality data. This correlation from a limited set of five genes investigated in the acute infection in BALB/c mouse point to low pH as probably the most unfavourable microenvironment (R = 0.9856) encountered by Mtb, among the various immune pressures (low pH, hypoxia, nutrient starvation, free radicals, etc.). However, similar studies using a larger set of genes under all the in-vitro physiologies as well as more relevant mouse models with human-like pathology may unleash these cidality correlations better.

Target *aroK*, like *rpoB*, the best in-vivo-cidal target, demonstrated a predominant cidality in the low pH screen (Table B in S1 File). It was interesting to discover that a seldom used, low pH screen, correlating best with in-vivo confirmation of cidal targets (*rpoB* and *aroK*); can statistically (R = 0.9856) predict in-vivo cidality (Fig 7). Does it mean that the replicating condition (7H9 media) and the rest of the in-vitro conditions are obsolete? May be/ maybe not. These correlations need to be verified in other mice strains like Kramnik that endows human like lung lesions and chronic disease.

Under the starvation of essential nutrients (Carbon, Nitrogen) in the granuloma, the non-replicating Mtb undergoes a global metabolic shift for energy conservation, shutting down some of its pathways, re-routing Carbon flow from central fatty acid metabolism to lipid and glutamate biosynthesis. Nitrogen is required in both replicating and non-replicating Mtb for biosynthesis of amino acids, nucleotides, organic cofactors to control critical molecular events (asparagine hydrolysis, ammonia release, pH buffering, growth) in the acidic environment, with α-ketoglutarate/glutamate as the key ‘nodal-point of pathways’[46–50]. In this study, most of the targets were cidal under in-vitro Nitrogen depletion condition (R = 0.4928), that enhances acid stress in granuloma; and restores the activity of lysosomal hydrolases to kill Mtb [46–50]. Thus, a “chicken or egg” conundrum, what triggers first? Are this Nitrogen depletion and low pH conditions, part of a vicious cycle; or, operate in tandem metabolically, along with other hostile conditions? Whatsoever, it requires a careful investigation and needs to be teased apart meticulously. However, difficult to crack, but it will be exciting and path-breaking.

The low pH, is probably subsidiary to another useful host defence mechanism, nitric oxide (NO, R = 0.5508), attacking Mtb with free radicals from innate immune cells; as the iNOS-/- (inducible NO synthase-deficient) mice, are reported to succumb to Mtb infection [4]. However, the cidality data from hypoxia or replicating growth conditions failed to correlate with in-vivo cidality outcome from BALB/c mouse model studies (Fig 7). Alternatively, it justifies the use of an appropriate in-vivo model that exhibits hypoxic lesions (not formed in BALB/c mouse) for better correlations.

Mtb infection in the BALB/c mouse leads to solid-granulomas harbouring primarily intracellular bacteria, but these lesions are not hypoxic and do not best represent lung pathology of human-TB [51]. Whereas, in the case of the human host, where mixes of both intracellular, as well as extracellular Mtb populations, exist, other conditions like hypoxia, and nutrient starvation may also play important roles. Though, the BALB/c mouse model has its limitations but is still better than investigating the in-vitro conditions alone.

In the recent years, development of several improvised gene knock-out mouse models has lead to better understanding of TB pathophysiology. These models display a human-like disease pathology in the lungs with hypoxia or other microenvironments of granuloma; harbouring mixed populations of Mtb: 1) Kramnik mice strains (C3HeB/FeJ [52]; 2). NOD-SCID/γc null of NSG model [53]; 3). iNOS-/- for NO synthase [4], etc. Each of these models has their pros and cons [54].

BALB/c mice have a strong precedence of use for testing drug efficacy including the investigational new drugs in the current pipeline, like TMC 207, PA824, SQ109, ADZ-5847, and Benzothiazinones, [55–60] and for target essentiality [61,62]. Hence, we used BALB/c mice strain in these studies. Although, BALB/c mice models are well validated for tuberculosis and are the standard route for translation in drug discovery, but, for cidality studies under different physiologies, it may not best represent the micro-environments and pathophysiology of human-TB. The solid granulomas harbouring primarily intracellular bacteria do not particularly develop hypoxia in BALB/c mice.

The low pH in-vitro screen emerging as the best-correlated predictor of in-vivo cidality, from BALB/c mouse model, may be a partial conclusion because of the two reasons: 1). Despite an effort to establish a hybrid of acute-chronic infection model in BALB/c, the high dose infection established represented mostly an acute-like infection with replicating bacteria analogous to early human disease. Hence, in the acute phase of infection for the high fidelity translation, the compound efficacy may also be evaluated in the low pH, in addition to the standard replication condition.2). The BALB/c mice used in the present study primarily form solid granuloma (only a single pathophysiological lesion type) harbouring largely the intracellular Mtb, unlike those observed (intra/extra-cellular Mtb with hypoxic microenvironment) in the cavitary lesions of human TB patients. Therefore, it is pertinent to perform such correlation studies using AS-recombinants of a larger set of target genes in humanised chronic infection models (Kramnik-C3HeB/FeJ) that may unleash these cidality correlations better. Kramnik model represents both extra/intra-cellular pathogen populations, along with most of the cavitary lesion types, as well as multiple stringent μ-environments of human-like granuloma that influence the pathogen to modulate its survival-specific genes for adaptation [51,54].

Our treatment therapy is effective on the acute phase of infection; it may not reflect what happens in the chronic phase. The low pH may not be important in general, as we earlier envisaged, but may be only relevant to the acute phase. The importance of the low pH or the appropriate screen in the chronic phase may be separately investigated.

Treatment of TB is complex; eradication of the dormant Mtb takes longer to treat due to hide-n-seek being played by this pathogen [63]. It cannot be attributed only to poor bio-availability of drugs [64,65], but may primarily be dictated by either the physiologic heterogeneity of bacteria in the tissues [66] or penetration of drugs in caseating foci/granuloma[66]. Rifampicin and PZA, the best sterilizing drugs responsible for shortening treatment duration in humans, penetrate better in to granulomas *vs*. moxifloxacin that largely concentrates in the periphery [66,67]. Interestingly all of these best tuberculocidals exhibit good MIC in acidic pH (Table D in S1 File), except isoniazid, which is inactive under acidic environment, hence, demonstrates a reduced activity on intracellular Mtb. The alarming statistics [1] on drug resistance in TB demand novel PZA-like sterilizing drugs that work best in low/acidic pH, kill non-replicating populations of Mtb and shorten the treatment duration [38,68]. In a separate study, we have demonstrated the importance of low pH screens by selecting PZA-hybrid molecules in-vitro [69]. Recently, PZA was also reported to enhance the cidality of various combinations of existing drugs [70]and is an integral component of emerging novel combinations in the clinic like PAMZ (PA-824, Moxi, PZA), in STAND (Shortening Treatment by Advancing New Drugs, www.clinicaltrial.com) clinical trial. Our findings unequivocally suggest that anti-TB activity in the low pH environment may be predictive of in-vivo tuberculocidality, especially for the acute phase of infection.

The low pH condition in-vivo appears to be a cumulative sum of various triggers and several secondary mechanisms. These triggers need to be explored in intricate details, and their appropriateness to identify tuberculocidal therapeutics may be strengthened further using large compound libraries and potential targets. The targets of stationary phase like *ppk*, need to be explored in the long-term infection models of experimental TB.

Our studies on in-vivo AS-repression of cidal targets lead to the killing of Mtb in-vitro and in-vivo, hence, demonstrating the bactericidal effect. It suggests that in the long run, AS-therapeutics can be explored in patients suffering from drug-sensitive and drug-resistant TB, for which currently there are only a few effective drugs available. It is a futuristic goal, but AS-therapeutics is an emerging radical approach to treating various diseases like anti-viral infections [71] (*Fomivirsen or Vitravene*) anti-cholesterol [72] (*mipomersen*), or even anti-cancer [73] (AP 12009) without any safety issues. However, as antibacterial, there is a need to develop improved AS-delivery systems for its successful applications. A lot of investigations are underway to overcome significant obstacles towards the development of efficient delivery systems like CPP (cell penetrating peptides), nanoparticles, or nanotubes, etc. [74,75] in the long process. Some companies are developing the AS delivery systems for bacterial therapeutic AS, ISIS Pharmaceuticals [ISIS Pharmaceuticals Inc.; now called Ionis pharmaceuticals http://www.ionispharma.com] is the leader in the field of AS-therapeutics.

An astute observation through the lens of antisense into a kaleidoscope of physiology-specific gene expression under in-vivo pressures in mouse; or, simulated in-vitro screens; unravelled a few secrets while a lot remain unrevealed.

Our studies with in-vivo AS-RNA-silencing of mycobacterial targets unravelled following features on target cidality: 1). *AroK* emerged as the in-vivo cidal target under diverse physiologies. These findings suggest *aroK* a potential target for developing mycobactericidal agents. 2). Use of *rpoB* target validated the entire concept on in-vitro to in-vivo translation. Like *rpoB*, *aroK* demonstrated that a druggable target needs to be cidal under all the physiological states in the host. 3). Cidality-SCORE is a rational approach to rank order cidality potential of the targets. Cidality-SCORE positively correlated in-vitro to the in-vivo translation of 4 out of 5 targets (*rpoB*, *aroK*, *rpoC*, and *ppK*) expressed under multiple physiologies. However, *ilvB* failed to translate to in-vivo cidality due to its auxotrophic nature and hence attenuation. 4). Low pH appeared to be an in-vivo predictor of cidality in acute infection. Hence, in the acute phase of infection for the high fidelity translation, the compound efficacy may also be evaluated in the low pH, in addition to the standard replication condition. Further, in-vitro to in-vivo translation correlations need to be studied in the chronic humanised models to narrow-down to a single in-vitro screen that alone can predict in-vivo (acute-chronic) cidality potential of the targets/ inhibitors.

We used a small subset of only five, potentially cidal target genes. Studies with a larger set of mixes of validated and unvalidated genes and their investigation in physiologically more relevant humanised mouse disease models like Kramnik and others are required to unravel other unrevealed shades.

## Supporting Information

S1 Fig Correlation of SPOT *vs*. cfu data of survival kinetics. (TIF)

S1 File “Table A in S1 File”, “Table B in S1 File”, “Table C in S1 File”, and “Table D in S1 File”.
(DOCX)
