# Supplementary figures and images for: Unravelling the Secrets of Mycobacterial Cidality through the Lens of Antisense
Source: PLoS One. 2016 May 4;11(5):e0154513. doi: 10.1371/journal.pone.0154513 (PMC4856384; doi:10.1371/journal.pone.0154513)

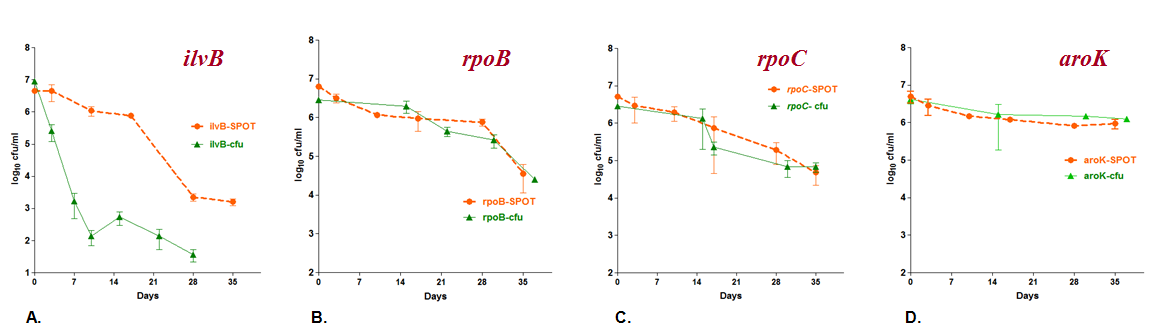

Supplement: S1 Fig — (TIF) [file pone.0154513.s001.tif]
