# Supplementary material for: Unravelling the Secrets of Mycobacterial Cidality through the Lens of Antisense
Source: PLoS One. 2016 May 4;11(5):e0154513. doi: 10.1371/journal.pone.0154513 (PMC4856384; doi:10.1371/journal.pone.0154513)
Supplement: S1 File — “Table A in S1 File”, “Table B in S1 File”, “Table C in S1 File”, and “Table D in S1 File”. (DOCX) [file pone.0154513.s002.docx]

**Supplemental Information:**

**Legends for the Supplementary figures**:

**Figure S1. Correlation of SPOT *vs.* cfu data of survival kinetics.** Genetically silenced targets demonstrated cidality dependent survival kinetics. (A). Target *ilvB* exceptionally depicted the cfu proximity dependent variability. Rest of the targets (B) *rpoB*, (C) *rpoC*, (D) *aroK* showed an excellent positive correlation hinting that SPOT assay can obviate the conventional laborious assay of plating.

**Legends for Supplementary Tables**:

**Table A. Primers used for RTPCR for bioavailability of AS-RNA.** In-vivo AS-RNA repression was tested by RTPCR of the net transcript available, using the Forward (F) and the Reverse (R) primers.

**Table B. AS silencing SCORE to represent the strength of a target.** The cidality SCORE in case of control strains shows a negative sign because these are growing, non-cidal. Rest of the recombinants have cidality-strength dependent positive numerals as SCOREs.

**Table C. PK of in-vivo effective AS-RNA gene silencing (RTPCR).** It is the target vulnerability dependent, and not the transcript-fold -repression based translation into cidality.

**Table D. MIC under different pH conditions (pH 6.2 & 7.2) with pH adapted Mtb cultures.** Pyrazinamide (PZA) and Rifampicin (Rif) are the best sterilizing drugs with best cidality under low pH conditions.

**Table A. Primers used for RTPCR for bioavailability of AS-RNA.**

| S.No. | Gene Primer | Primer sequence |
| --- | --- | --- |
| 1 | *aroK* RT (F1) | 5’-atggcacccaaagcggttctcg-3’ |
| 2 | *aroK* RT (R1) | 5’-acaccgtcgtggtcggccagtg-3’ |
| 3 | *rpoC* RT (F) | 5’-tgtactacctgaccaccgagg-3’ |
| 4 | *rpoC* RT (R) | 5’-ttcttgtgcatctgcttgttg-3’ |
| 5 | *ppk* RT (F) | 5’-ttacaacagcaagacagcacg-3’ |
| 6 | *ppk* RT (R) | 5’-catcaacaagggcattcatct-3’ |
| 7 | *ilvB* RT (F) | 5’- ttcatcagatacgaaaagccg-3’ |
| 8 | *ilvB* RT (R) | 5’-catagaacaggctctgccact-3’ |
| 9 | *rpoB* RT (F) | 5’-ctgttgaccatcaagtccgat-3’ |
| 10 | *rpoB* RT (R) | 5’-ttacgcaagatcctcgacact-3’ |

**Table B. AS silencing SCORE to represent the strength of a target.**

| r-Mtb  AS | Target | Essential/non-Essential | SCORE of cfu reduction (log_10_/ml) | | | | | | | Outcome |
| --- | --- | --- | --- | --- | --- | --- | --- | --- | --- | --- |
| Strain | Function | Sassetti | REP | Hpx | NSM | NO | LpH | Msx | Score | Better cidality SCORE in |
| WT | Control strain | WT | -1.83 | -0.61 | -0.27 | -0.28 | -0.67 | -0.50 | -4.2 | Growing as expected |
| V | Control strain | EV | -1.75 | -0.56 | -0.22 | -0.56 | -0.39 | -0.27 | -3.8 | Growing as expected |
| *rpoB* | Transcription | E | 2.24 | 0.75 | 0.83 | 0.38 | 1.42 | 1.44 | 7.1 | Low pH, N2 |
| *rpoC* | Transcription | E | 2.03 | 0.63 | 0.66 | 0.18 | 0.41 | 1.33 | 5.2 | REP, N2 |
| *aroK* | Aromatic aa | E | 0.71 | 0.44 | 0.56 | -0.03 | 0.76 | 0.72 | 3.2 | Low pH, N2 |
| *ppk1* | Long chain poly-phosphate | E | 3.08 | 1.42 | 0.74 | 0.22 | 0.45 | 1.64 | 7.5 | REP |
| *ilvB* | Branched aa | E | 3.46 | 1.11 | 1.07 | 0.16 | 0.35 | 1.16 | 7.3 | REP |

**Table C. PK of in-vivo effective AS-RNA gene silencing (RTPCR).**

| S. No | Mtb GENE/ strain | Maximum fold-transcript reduction (day) | Maximum cfu log_10_ reduction | Significant  (2-way ANOVA) P<0.05 | Comments:  Transcript vs. cidality |
| --- | --- | --- | --- | --- | --- |
| 1 | WT Mtb | 1 | 0 | Control | Control |
| 2 | Vector | 1 | 0.2 | ns | No significant difference from the WT Mtb |
| 3 | *ilvB* | 31.1 (42) | 0.36 | ** | Probably due to auxotrophy and atttenuation ? |
| 4 | *rpoC* | 43.4 (7) | 1.6 | *** | ~40 fold AS repression is also not enough to translate into in-vivo cidality |
| 5 | *ppk* | 103.6 (3) | 1.6 | *** | Even ~100-fold AS repression is not enough to show in-vivo cidality |
| 6 | *aroK* | 39.7 (3) | 2.4 | *** | Target is in-vivo cidal and vulnerable |
| 7 | *rpoB* | 13.2 (28) | 3.9 | *** | Target is highly in-vivo cidal/vulnerable. This is a clinically proven target. |

**Table D. MIC under different pH conditions (pH 6.2 and 7.2).**

|  | MIC (ug/ml) | | | | | | | | |
| --- | --- | --- | --- | --- | --- | --- | --- | --- | --- |
| pH | Str | Inh | Rif | Emb | Oflox | Moxi | Cipro | Roxi | PZA |
| 6.2 | 4-16 | >4 | 0.06 | 4 | 2 | 2 | 2-4 | 16 | 64-128 |
| 7.2 | 0.5-1 | 0.03 | 0.015 | 0.5 | 0.25 | 0.25 | 0.5 | 2 | >256 |
